# PGE2 Augments Inflammasome Activation and M1 Polarization in Macrophages Infected With *Salmonella* Typhimurium and *Yersinia enterocolitica*

**DOI:** 10.3389/fmicb.2018.02447

**Published:** 2018-10-31

**Authors:** Austin E. F. Sheppe, Evangel Kummari, Alyssa Walker, Angela Richards, Winnie W. Hui, Jung Hwa Lee, Lauren Mangum, Abdolsamad Borazjani, Matthew K. Ross, Mariola J. Edelmann

**Affiliations:** ^1^Department of Microbiology and Cell Science, College of Agricultural and Life Sciences, University of Florida, Gainesville, FL, United States; ^2^Department of Basic Sciences, Center for Environmental Health Sciences, College of Veterinary Medicine, Mississippi State University, Starkville, MS, United States

**Keywords:** eicosanoids, *Yersinia enterocolitica*, *Salmonella enterica* Typhimurium, inflammasome, macrophage polarization

## Abstract

Eicosanoids are cellular metabolites, which shape the immune response, including inflammatory processes in macrophages. The effects of these lipid mediators on inflammation and bacterial pathogenesis are not clearly understood. Certain eicosanoids are suspected to act as molecular sensors for the recruitment of neutrophils, while others regulate bacterial uptake. In this study, gene expression analyses indicated that genes involved in eicosanoid biosynthesis including COX-1, COX-2, DAGL, and PLA-2 are differentially regulated in THP-1 human macrophages infected with *Salmonella enterica* Typhimurium or *Yersinia enterocolitica*. By using targeted metabolomics approach, we found that the eicosanoid precursor, arachidonic acid (AA) as well as its derivatives, including prostaglandins (PGs) PGF2α or PGE2/PGD2, and thromboxane TxB2, are rapidly secreted from macrophages infected with these Gram-negative pathogenic bacteria. The magnitude of eicosanoid biosynthesis in infected host cells depends on the presence of virulence factors of *Y. enterocolitica* and *S.* Typhimurium strains, albeit in an opposite way in *Y. enterocolitica* compared to *S.* Typhimurium infection. Trials with combinations of EP2/EP4 PGE2 receptor agonists and antagonists suggest that PGE2 signaling in these infection models works primarily through the EP4 receptor. Downstream of EP4 activation, PGE2 enhances inflammasome activation and represses M2 macrophage polarization while inducing key M1-type markers. PGE2 also led to a decreased numbers of *Y. enterocolitica* within macrophages. To summarize, PGE2 is a potent autocrine/paracrine activator of inflammation during infection in Gram-negative bacteria, and it affects macrophage polarization, likely controlling bacterial clearance by macrophages.

## Introduction

**FIGURE 1:**
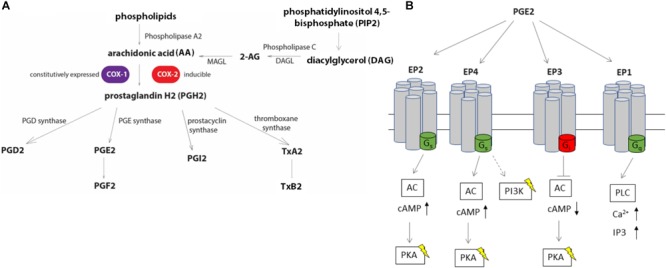
Eicosanoid biosynthesis and PGE2 signaling *via* EP receptors. **(A)** Biosynthesis of eicosanoids relies on the release of free arachidonic acid (AA) from membrane phospholipids *via* phospholipase A2 (PLA-2) or conversion of diacylglycerol to AA through phospholipase C (PLC). An alternative source of AA includes 2-arachidonoylglycerol (2-AG). AA is converted to prostaglandin H2 (PGH2) *via* the action of COX enzymes, COX-1 and COX-2. PGH2 can be converted to other derivatives, including PGE2 by PGE synthases. **(B)** PGE2 stimulates at least four different G-protein coupled receptors (EP1, EP2, EP3, and EP4). Stimulation of EP2 and EP4 receptors lead to an increase in intracellular cAMP levels by conversion of ATP to cAMP by adenylate cyclase (AC), while EP3 decreases intracellular cAMP levels by binding to the inhibitory G-protein subunit to AC upon PGE2 stimulation. The EP4 receptor stimulates PI3K pathway independent of cAMP, such as by modulating gene expression *via* NF-κB. EP1 activity is linked to the mobilization of intracellular Ca^2+^ by activating the phospholipase C pathway.

*Salmonella enterica* Typhimurium (*S.* Typhimurium) and *Yersinia enterocolitica* are Gram-negative bacteria and significant causative agents of foodborne infections arising from contaminated food sources. Societal costs of *S.* Typhimurium and *Y. enterocolitica* infections account for several billion US dollars annually (Scharff, 2012; Hoffmann and Anekwe, 2013). Innate immune responses play an essential role in the host response to *S.* Typhimurium infection, but many details remain unanswered, such as how host metabolites activate the innate immune responses for bacterial clearance. Metabolomic studies identified changes in host metabolites upon *S.* Typhimurium infection, suggesting that metabolites of one of the groups, eicosanoids are elevated during infection in a murine model (Antunes et al., 2011; Deatherage Kaiser et al., 2013). The eicosanoid pathway (Figure 1), which depends on the activity of cyclooxygenase (COX) enzymes (Tanioka et al., 2000), is known to be affected by infections with bacterial pathogens (Antunes et al., 2011; Deatherage Kaiser et al., 2013; Alugubelly et al., 2016). For instance, COX-2 is upregulated in murine macrophages infected with *S.* Typhimurium, which depends on the presence of the *Salmonella* Pathogenicity Island-2 (SPI-2) SpiC protein (Uchiya and Nikai, 2004). Furthermore, a metabolic profiling study in animal tissue identified upregulation of the PGD2 metabolite 15-deoxy-Δ^12,14^-PGJ2 upon *Salmonella* infection (Antunes et al., 2011). This eicosanoid was shown to successfully prevent bacterial colonization during *Salmonella* infection of mouse and human macrophages (Buckner et al., 2013). Following activation of the enzymes responsible for eicosanoid biosynthesis, these metabolites can either trigger or prevent immune responses (Funk, 2001). For instance, prostaglandins, leukotrienes, and 15-hydroxyeicosatrienoic acid (HETE), 15-HETE, and 12-HETE are rapidly produced during the activation of inflammasome (von Moltke et al., 2012; Rauch et al., 2017). Eicosanoids are also involved in neutrophil recruitment, for instance, leukotriene LTB4 serves as a chemoattractant for these cells (Lämmermann et al., 2013; Tyrkalska et al., 2016). Finally, prostaglandin E2 (PGE2) is involved in gut wound repair (Jackstadt and Sansom, 2017; Zhuang et al., 2017) and is released upon inflammasome activation (von Moltke et al., 2012; Dennis and Norris, 2015). However, the function of eicosanoids such as PGE2 in inflammasome activation remains controversial, and their role in the clearance of Gram-negative infections by phagocytic cells remains vastly unknown. Considering the variety of processes mediated by PGE2 and other eicosanoids in different physiological models, our goal was to determine the function of these bioactive lipids in human macrophages in response to *S.* Typhimurium and *Y. enterocolitica* infections. *S.* Typhimurium infection leads to an inflammasome activation in infected macrophages, which is controlled by *Salmonella* Pathogenicity 1 and 2 (SPI-1 and SPI-2) effectors.

By contrast, the protein effectors encoded within the virulence plasmid of *Y. enterocolitica* prevent inflammasome formation and support bacterial survival within the macrophage (Brodsky et al., 2010). Deletion of a virulence plasmid from *Y. enterocolitica* leads to an increased release of eicosanoids from infected macrophages while impairment of SPI-2 in *S.* Typhimurium causes an attenuation of eicosanoid release from macrophages in comparison to the wild-type (wt) *S.* Typhimurium infection. One of these eicosanoids which were increasingly released from macrophages infected with *S.* Typhimurium or *Y. enterocolitica* was PGE2. We showed that PGE2 leads to an increased IL-1β production and release from macrophages *via* EP4- and EP2 receptor signaling pathways. We also demonstrate that PGE2 increases specific pro-inflammatory cytokines relevant to infection and characteristics of M1 phenotype (mainly IL-1β and IL-23/17) while shifting polarization away from the M2 phenotype. Finally, we show that PGE2 affects the number of *Y. enterocolitica* within macrophages.

## Materials and Methods

### Cell Culture Conditions

THP-1 monocytic cells (ATCC # TIB-202, ATCC, United States) were cultured in RPMI 1640 (Gibco, Life Technologies Inc., United States) supplemented with 10% fetal bovine serum (FBS), 2 mM Glutamax (Gibco, Life Technologies Inc., United States), and 100 μg/mL penicillin/streptomycin (Life Technologies Inc., United States) in a humidified atmosphere of 5% CO_2_ at 37°C. For activation and differentiation of THP-1 cells into macrophages, phorbol 12-myristate 13-acetate (PMA; Sigma-Aldrich, United States) was used (100 nM), and cells were incubated for 24 h. This particular cell line has been chosen because it has already been well characterized in the *Y. enterocolitica* and *S. enterica* infection models (Winter et al., 2015; Chung et al., 2016). For instance, the level of IL-1β secretion has been shown to be comparable to the one obtained in human peripheral blood mononuclear cells, which is relevant since in our study we are particularly interested in the inflammasome activity (Chung et al., 2016).

*Salmonella enterica* serovar Typhimurium wt ATCC 14028 and isogenic Δ*ssaV* mutant [a generous gift from Dr. David Holden (Beuzón et al., 2000)], or *S*. Typhimurium UK-1 χ3761 wt and its isogenic mutants (gifts from Dr. Roy Curtiss III and Qingke Kong, University of Florida; Table 1), were cultured in LB at 37°C at 200 rpm (MaxQ 4450 incubator, Thermo Scientific, United States). Overnight cultures were split to achieve OD_600_ = 0.05 and cultured using the same conditions until OD_600_ reached 0.5 (early exponential phase), centrifuged at 5,000 × *g*, washed with phosphate-buffered saline (PBS) and resuspended in cell culture medium for infections.

**Table 1:** Strains used in this study.

Strain	Phenotype/genotype	Reference
*Salmonella enterica* ser. Typhimurium ATCC 14028 wt	wt	Beuzón et al., 2000
*Salmonella enterica* ser. Typhimurium ATCC 14028 Δ*ssaV*	Impaired SPI-2	Beuzón et al., 2000
*Salmonella enterica* ser. Typhimurium UK-1 x3761 wt	wt	Hassan and Curtiss, 1990
*Salmonella enterica* ser. Typhimurium UK-1 x11754	*ΔaroA*	Kong et al., 2011
*Salmonella enterica* ser. Typhimurium UK-1 x11312	Rough LPS, *ΔwaaL46*	Kong et al., 2011
*Salmonella enterica* ser. Typhimurium UK-1 x12253	Rough LPS, *ΔwaaC41*	Liu et al., 2016
*Yersinia enterocolitica* 8081 pYV wt	wt	Portnoy et al., 1981
*Yersinia enterocolitica* 8081c	Deletion of a virulence plasmid	Portnoy et al., 1981

*Yersinia enterocolitica* 8081 wt (pYV) and its isogenic virulence plasmid-cured mutant (8081c) strain [a generous gift from Dr. James Bliska (Portnoy et al., 1981); Table 1] were grown in LB overnight (18 h) at 27°C. The culture was diluted as above to achieve OD_600_ 0.05 and incubated at 27°C for approximately 2 h until OD_600_ reached 0.25. The temperature was then changed to 37°C to activate the type three secretion system (TTSS), and bacteria were grown until OD_600_ reached 0.5, centrifuged at 5,000 × *g*. Bacteria were centrifuged at 5,000 × *g*, washed with PBS, and resuspended in cell culture medium for infections.

### Infections and Inflammasome Activation

THP-1-derived macrophages were washed with PBS and incubated in RPMI media containing 10% FBS and no antibiotics for 60 min before infection. Bacteria were also washed with PBS, resuspended in RPMI media containing 10% FBS, and used to infect cells (MOI of 50:1) for times indicated in figures, after which the cell culture supernatant and cells were collected, centrifuged once to remove bacteria, and then the supernatant was recovered. The uninfected control cells were treated in the same way as the infected cells, but no bacteria were added.

THP-1 cells were incubated with *S.* Typhimurium lipopolysaccharide (LPS) [(1 μg/ml); Sigma-Aldrich] for 4 h, followed by treatment with nigericin (10 μM) for 1 h, similarly as published previously (Kummari et al., 2015) to activate inflammasome. In cells treated with a UCH-L5/USP14 inhibitor, the THP-1 macrophages were treated with (1 μM) b-AP15 (or vehicle control, DMSO added at equal volume to a final concentration of 0.01% v/v) for 15 min, followed by 2-h infection with *S.* Typhimurium (MOI 50:1).

### Transcript Analysis by RT-qPCR

RNA was extracted from cells by using Qiagen RNeasy Mini Plus extraction kit. Next, cDNA was generated using Maxima First Strand cDNA synthesis kit (Thermo Fisher) and expression of genes COX-1, COX-2, DAGL, MGGL (MAGL), PLA-2, and TXBSA was measured by using two-step quantitative real-time polymerase chain reaction (RT-qPCR), performed on a Stratagene MXP3005 using SYBRGreen reagents (Bio-Rad). Fold change in gene expression was estimated by using the ΔΔCt method, and statistical significance was determined as previously described (Schmittgen and Livak, 2008). For analysis of mRNA levels of PGE2 receptors, SOCS3, MRS1, and COX-2, RNA was extracted from cells by using a PureLink RNA Mini Kit (Thermo Fisher). The cDNA was synthesized using a Bio-Rad iScript cDNA Synthesis Kit and expression of genes encoding EP1, EP2, EP3, and EP4 was measured as stated above on a Bio-Rad CFX96 Real-Time System. Fold change and statistical significance were determined (Schmittgen and Livak, 2008), and the expression data were normalized to the expression of RPL37A. Primers used for the study were validated bioinformatically and by using melt curve analysis (Table 2). Additionally, we performed western blotting by using antibodies raised against select proteins (anti-COX-2 and anti-PLA-2, Santa Cruz Biotechnology, United States).

**Table 2:** Primers used in RT-PCR studies.

Oligo name	Sequence
Human EP1 forward	ATGGTGGGCCAGCTTGTC
Human EP1 reverse	GCCACCAACACCAGCATTG
Human EP2 forward	CTGCTGCTGCTTCTCATTGT
Human EP2 reverse	ATGCGGATGAGGTTGAGAAT
Human EP3 forward	GGTCTCCGCTCCTGATAATG
Human EP3 reverse	CTCCGTGTGTGTCTTGCAGT
Human EP4 forward	CGACCTTCTACACGCTGGTATG
Human EP4 reverse	CCGGGCTCACCAACAAAG
Human MSR1 forward	CCGGAAGGCCAGGAAATTCT
Human MSR1 reverse	CCACCGACCAGTCGAACTTT
Human SOCS3 forward	ATTCGGGACCAGCCCCC
Human SOCS3 reverse	AACTTGCTGTGGGTGACCAT
Human MRC1 forward	CGATCCGACCCTTCCTTGAC
Human MRC1 reverse	TGTCTCCGCTTCATGCCATT
Human TGM2 forward	ATAAGTTAGCGCCGCTCTCC
Human TGM2 reverse	ACCAGCTCCTCGGCCAT
Human MSR1 forward	CCGGAAGGCCAGGAAATTCT
Human MSR1 reverse	CCACCGACCAGTCGAACTTT
Human MCP1 forward	CGCTCAGCCAGATGCAATCAA
Human MCP1 reverse	GACACTTGCTGCTGGTGATTC
Human iNOS forward	TCCCGAAGTTCTCAAGGCAC
Human iNOS reverse	TTCTTCACTGTGGGGCTTGC
Human SOCS3 forward	ATTCGGGACCAGCCCCC
Human SOCS3 reverse	AACTTGCTGTGGGTGACCAT
Human HPRT1 forward	CCCTGGCGTCGTGATTAGT
Human HPRT1 reverse	TCTCGAGCAAGACGTTCAGT
Human TBP forward	AGTGACCCAGCATCACTGTTT
Human TBP reverse	GAGCATCTCCAGCACACTCT
Human RPL37A forward	ATTGAAATCAGCCAGCACGC
Human RPL37A reverse	AGGAACCACAGTGCCAGATCC
Human GAPDH forward	AAGGTGAAGGTCGGAGTCAACG
Human GAPDH reverse	CCTTCTCCATGGTGGTGAAGAC
Human CD200R forward	ATGCTCTGCCCTTGGAGAAC
Human CD200R reverse	CTCCGCTTCGGCCACTAA
Human CCL17 forward	AGGGAGCCATTCCCCTTAGA
Human CCL17 reverse	GCACAGTTACAAAAACGATGGC
Human MMP12 forward	AACCAACGCTTGCCAAATCC
Human MMP12 reverse	CCTTCAGCCAGAAGAACCTGT
Human NOX2 forward	TTCACTCTGCGATTCACACCA
Human NOX2 reverse	CGGGCATTCACACACCATTC
Human IL-1b forward	TGAGCTCGCCAGTGAAATGA
Human IL-1b reverse	AGATTCGTAGCTGGATGCCG
Human IL-12B forward	GCCCAGAGCAAGATGTGTCA
Human IL-12B reverse	CACCATTTCTCCAGGGGCAT

### Extraction of Media for Eicosanoid Analysis

Hypersep Retain Pep columns (Thermo Scientific, United States) were used to extract eicosanoids from the media. The columns were first activated with ethyl acetate and methanol, and then equilibrated with 95:5 water:methanol containing 0.1% acetic acid. The cell culture supernatants were added to the column along with PGF2α-d4, AA-d8, and 2AG-d8 as internal standards. The column was washed with 95:5 water:methanol containing 0.1% acetic acid and the eicosanoids were eluted in methanol and ethyl acetate and dried under nitrogen. The dried precipitate was dissolved in 1:1 methanol:water and used for eicosanoid analysis.

### Eicosanoid Analysis by Triple Quadrupole Mass Spectrometry

The media extracts were analyzed by targeted metabolomics approach for AA, PGF_2α_, PGE_2_, PGD_2_, and TXB_2_. Acquity UPLC system (Waters, Milford, MA, United States) coupled to a TSQ Quantum Access triple-quadrupole tandem mass spectrometer equipped with a heated electrospray (H-ESI) source (Thermo Fisher Scientific, San Jose, CA, United States) was used for metabolomics studies. Chromatographic separation was carried out using an Acquity UPLC BEH C18 column (2.1 mm × 100 mm, 1.7 μm) equipped with a VanGuard precolumn (2.1 mm × 5 mm, 1.7 μm) at 40°C using a column oven and 10 μL of samples were injected into the system. The mobile phases used were water with 0.1% acetic acid (A) and methanol with 0.1% acetic acid (B). Mobile phage gradient conditions were as follows: hold at 85% A and 15% B for 1 min, linear increase of B to 80% in 11 min, hold at 80% B for 1 min, increase of B to 100% in 0.5 min, hold at 100% for 1.5 min, decrease of B to 15% in 0.5 min and equilibrate for 4.5 min at the starting conditions. The overall run time was 20 min, and the flow rate was 0.2 mL/min. The eluate from the LC was directly electrosprayed into mass spectrometer using an electrospray ionization interface in negative mode. MS conditions were set as follows: spray voltage = 4000 V, vaporizer temperature = 400°C, sheath gas = 25 psi, auxiliary gas = 2 psi, and capillary temperature = 320°C. Samples were run in selected reaction monitoring (SRM) mode and precursor-to-product ion transitions *m*/*z* 303.2 →*m*/*z* 259.3 for AA, *m*/*z* 353.2 →*m*/*z* 193.2 for PGF_2α_, *m*/*z* 351.3 →*m*/*z* 271.2 for PGE and PGD_2_, and *m*/*z* 369.2 →*m*/*z* 169.1 for TXB_2_. Internal standards included *m*/*z* 311.2 →*m*/*z* 267.2 for AA-d_8_ and *m*/*z* 357.3 → m/z 197.2 for 8-iso-PGF_2α_-d_4_, which were used to quantify the amounts of AA and PGs. Scan time was 0.2 s per SRM, and the scan width was *m*/*z* 0.01. Optimum collision energy and S-lenses conditions were determined for each compound by using auto-tune software for each analyte by post-column infusion of the individual compounds into a 50% A/50% B blend of the mobile phase being pumped at a flow rate of 0.2 mL/min. Xcalibur software was employed for data acquisition and processing.

### Quantification of PGE2 by ELISA

Prostaglandin E2 ELISA-monoclonal (Item No. 514010, Cayman Chemical Company, United States) was performed for quantification of PGE2 in cell culture supernatants per the manufacturer’s recommendations. The plate was read by using a Cytation3 imaging plate reader (BioTek, VT, United States).

### Treatment With EP2 and EP4 Receptor Agonists/Antagonists and yVAD-CHO

A total of 250,000 THP-1 cells per well were seeded in 24-well plates and differentiated for 24 h by using PMA (100 nM). One hour before infection, cells were washed, and complete cell culture medium lacking antibiotics were added, which were supplemented with EP4/EP2 receptor agonists/antagonists or appropriate vehicle control (EtOH and DMSO, depending on the treatment). The following EP2 or EP4 agonists and antagonists were used: PGE2 [(2 μM) or as indicated], EP2 receptor agonist Butaprost (10 μM), EP2 receptor antagonist PF-04418948 (200 nM), EP4 receptor agonist L902,688 (1 μM), EP4 receptor antagonist L-161,982 (200 nM), and YVAD-CHO (1 μM). All chemicals, including PGE2, were purchased from Cayman Chemicals (MI, United States). Following the treatment, cells were infected with *S.* Typhimurium or *Y. enterocolitica* at an MOI of 50:1 for 2 h or as indicated. The cell supernatant was centrifuged at 500 × g for 5 min, and IL-1β was quantified in the supernatant by using ELISA (R&D Systems). Cytotoxicity was measured by using the LDH assay (Thermo Scientific) which was performed according to the manufacturer’s instruction.

### Gentamicin Protection Assay

THP-1-derived macrophages were treated with PGE2 or vehicle control (ethanol, 0.01% v/v). PGE2 or vehicle control was added to complete RPMI cell culture medium lacking antibiotics 2 h before infection with *Salmonella* Typhimurium (MOI of 50:1). Thirty minutes post-infection, media were removed, and cells were washed twice with PBS to remove extracellular bacteria. RPMI media lacking antibiotics supplemented with gentamicin (100 μg/mL), +/-PGE2 were added onto cells, and cells were incubated further. Two hours post-infection (hpi), media were removed again, cells washed twice with PBS, and resuspended in media containing a lower concentration of gentamicin (10 μg/mL) for the remainder of the infection. At the indicated time points, cells were washed, lysed with 0.1% Triton-X, diluted in sterile PBS, and plated on LB plates for CFU counts. A similar protocol was performed for *Y. enterocolitica* infection, based on our past work (Edelmann et al., 2010; Alugubelly et al., 2016).

### Confocal Microscopy

THP-1 macrophages (1.5 × 10^6^) were pre-treated with PGE2 (2 μM) or equal (v/v %) concentration of vehicle control (ethanol; final concentration was 0.01%) for 2 h prior to infection with wt *S.* Typhimurium (MOI of 50:1, 48 hpi). Cells were fixed, permeabilized, and stained for the actin cytoskeleton (ActinRed 555) and nucleus (DAPI stain). The cells were visualized by using a Confocal Zeiss LSM800 microscope and data were analyzed using Zen (Blue Edition) software. The polarization of cells was determined by measuring cell dimensions since the elongation of human macrophages indicates their M1 type polarization (Zajac et al., 2013). Elongated cells were determined as having a length to diameter ratio of at least 2:1. Approximately 150 cells were counted across five biological replicates for each treatment.

### Statistical Analysis

Statistical analysis was performed by using GraphPad Prism. Student’s *t*-test was used with a 95% confidence interval (minimum acceptable *p*-value was 0.05). Alternatively, we used ANOVA tests in conjunction with Tukey’s multiple comparison tests (*p* < 0.05) as indicated on figures.

### Institutional Safety Procedures

Accidental exposure to pathogenic bacteria here described can cause gastroenteritis and enterocolitis. Standard BSL2 practices were followed, and personnel was carefully advised about biohazards and ways to minimize the chances of exposure. The study has been completed by following standard operating procedures outlined for this project.

## Results

### Changes in Expression of Genes Regulating Eicosanoid Pathway Upon Infection With Gram-Negative Bacteria

**FIGURE 2:**
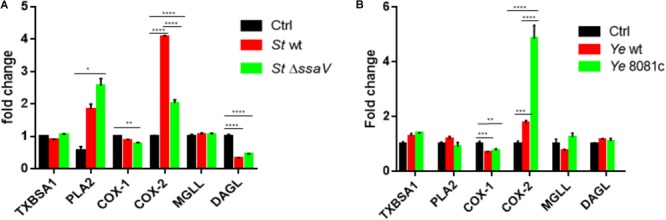
Eicosanoid biosynthetic genes are upregulated upon infection with *S.* Typhimurium or *Y. enterocolitica*. Targeted RT-PCR of eicosanoid biosynthetic genes was performed on THP-1-derived macrophages either uninfected/infected with *S.* Typhimurium **(A)** or *Y. enterocolitica* **(B)** for 2 h at an MOI of 50:1. For comparison, isogenic strains of *S.* Typhimurium lacking the T3SS (Δ*ssaV)* and *Y. enterocolitica* lacking the pYV virulence plasmid (8081c) were used to infect THP-1 cells. Two-step RT-PCR was performed on a Stratagene MXP3005 using SYBRGreen reagents. Fold change was calculated using the ΔΔCt method. Two-way ANOVA along with Tukey’s *post hoc* tests were used to calculate significance (*N* = 3). *p*-values were indicated as follows: ^∗^*p* ≤ 0.05; ^∗∗^*p* ≤ 0.01; ^∗∗∗^*p* ≤ 0.001; ^∗∗∗∗^*p* ≤ 0.0001.

We hypothesized that the expression of specific genes regulating eicosanoid biosynthesis in human macrophages is modulated at early stages of *S.* Typhimurium and *Y. enterocolitica* infections (2 hpi). We measured transcripts of select genes regulating eicosanoid biosynthesis in infected or uninfected cells by using quantitative RT-PCR. The most significantly upregulated transcript in *S.* Typhimurium-infected macrophages was COX-2 (Figure 2A). Disruption of SPI-2 by *ssaV* deletion from *S.* Typhimurium led to downregulation of COX-2 in macrophages, and upregulation of PLA-2 in comparison to wt *S.* Typhimurium-infected cells (Figure 2A), suggesting that proteins encoded within SPI-2 play a key role in upregulation of COX-2, but not of other tested transcripts. COX-1 transcripts were slightly downregulated upon Δ*ssaV S.* Typhimurium infection in comparison to control. Finally, diacylglycerol lipase (DAGL) transcript was significantly downregulated in cells infected by both wt and Δ*ssaV S.* Typhimurium in comparison to uninfected control. DAGL is an essential component of the endocannabinoid system, hydrolyzing diacylglycerol to 2-arachidonoylglycerol (2-AG), where 2-AG can serve as an alternative pathway to obtain prostaglandin precursors. Importantly, in an attempt to validate the transcript data we performed western blotting analysis of COX-2 and PLA-2 proteins in cell lysates from infected macrophages (Supplementary Figure S1A).

*Y. enterocolitica* infection led to an upregulation of COX-2 gene expression in infected macrophages, and downregulation of COX-1, while other changes in transcripts were not statistically significant. In cells infected with virulence plasmid-cured *Y. enterocolitica* (8081c), COX-2 gene expression was increased in comparison to wt *Y. enterocolitica* infections (Figure 2B). *Y. enterocolitica*’s virulence plasmid pYV was therefore shown to play a role in dampening expression of COX-2, although specific virulence factors encoded within this plasmid affecting COX-2 expression remain to be identified.

### Production of Eicosanoids by Macrophages Upon Infection With *S.* Typhimurium and *Y. enterocolitica*

**FIGURE 3:**
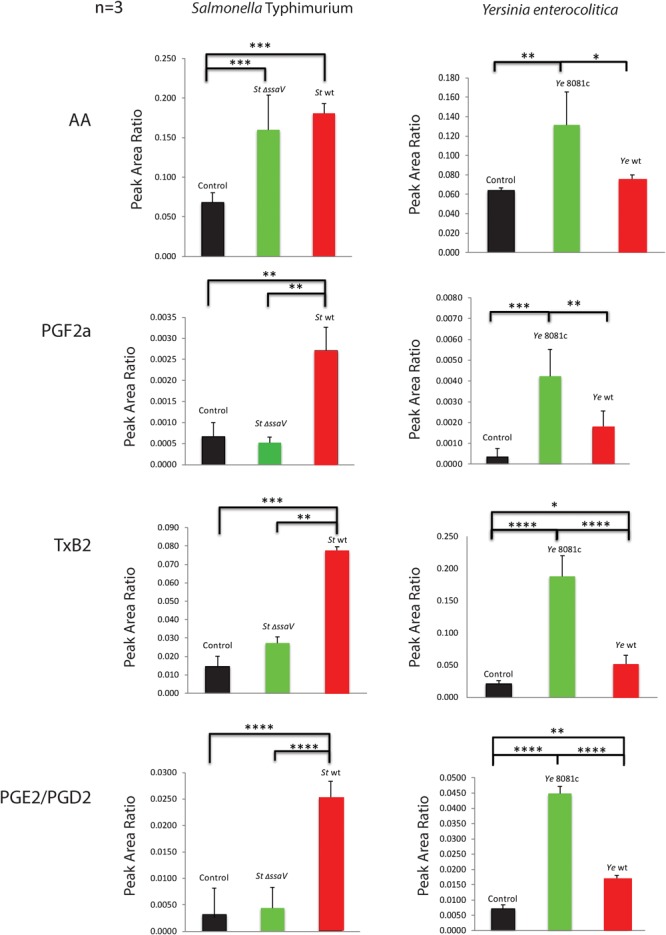
Levels of eicosanoids secreted into culture medium by macrophages infected with *S.* Typhimurium or *Y. enterocolitica*. Triple quadrupole mass spectrometry-based targeted metabolomics study was performed on cell culture supernatants of THP-1 macrophages infected with *S.* Typhimurium or *Y. enterocolitica* for 2 h at an MOI of 50:1. The media extracts were analyzed for AA, PGF_2α_, TxB2, and PGE2/PGD_2_, and relative abundance was calculated using internal standards (*N* = 3). Significance was calculated by using Student’s *t*-test. *p*-values were indicated as follows: ^∗^*p* ≤ 0.05; ^∗∗^*p* ≤ 0.01; ^∗∗∗^*p* ≤ 0.001; ^∗∗∗∗^*p* ≤ 0.0001.

Triple quadrupole quantitative mass spectrometry was next used to quantify the levels of eicosanoids released by infected macrophages. AA, PGF2α, TxB2, and PGE2/PGD2 were increasingly released from macrophages upon wt *S.* Typhimurium infection (Figure 3), while infection with Δ*ssaV S.* Typhimurium resulted in the lower release of PGF2α, PGE2/PGD2, and TxB2 in comparison to wt *S.* Typhimurium. In comparison to uninfected cells, Δ*ssaV S.* Typhimurium infection still led to significant upregulation of AA and TxB2 eicosanoids in cell culture medium, but not PGF2α or PGE2/PGD2. Macrophages infected with *Y. enterocolitica* infection virulence plasmid-cured (8081c) or wt strain, produced an increased amount of all targeted eicosanoids (AA, PGF2α, and PGE2/PGD2 but also, albeit less significantly of TxB2). Furthermore, deletion of the pYV plasmid controlling the expression of effector proteins of *Yersinia* led to an increased release of AA, TxB2, and PGE2/PGD2 from infected macrophages in comparison to wt *Y. enterocolitica* infection. Moreover, cells pretreated with b-AP15 inhibitor, which downregulates inflammasome *via* inhibition of specific deubiquitinating enzymes (Crump et al., 2015), blocked the production of *S.* Typhimurium-induced eicosanoid production (Crump et al., 2015) (Supplementary Figure S1B). Finally, inflammasome activation by using treatment with *S.* Typhimurium-derived LPS and nigericin led to a release of PGE2/PGD2 and TxB2 from macrophages (Supplementary Figure S1C), consistent with other published work (von Moltke et al., 2012; Dennis and Norris, 2015).

### PGE2 Affects Bacterial Load During Infection With *Y. enterocolitica* but Not *S.* Typhimurium Infection

**FIGURE 4:**
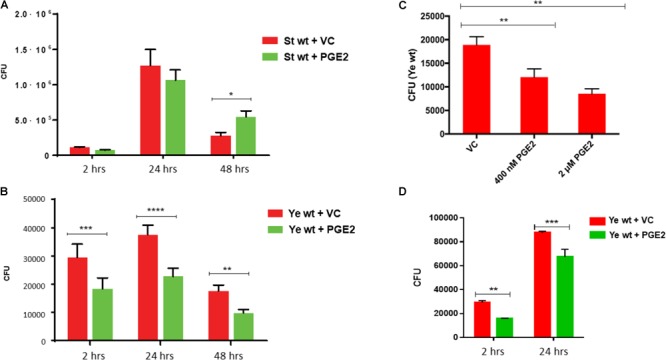
Effect of PGE2 on bacterial load during infection with *Salmonella* Typhimurium or *Yersinia enterocolitica*. THP-1 macrophages (2.5 × 10^5^) were infected with **(A)** wild-type *Salmonella* or **(B), (C)** wild-type *Yersinia* (MOI of 50:1) in the presence or absence of PGE2. At the indicated times post-infection, macrophages were lysed with 0.1% Triton-X, diluted, and dilutions were plated on LB agar plates. A dose–response curve was generated by pretreating THP-1 macrophages with varying concentrations of PGE2 before infection with *Y. enterocolitica*, and then the resulting CFUs were measured 2 hpi **(C)**. Similarly to **(B)**, uptake and survival of *Y. enterocolitica* in HeLa cells was also evaluated **(D)**. Results are represented as the mean ± SD of at least three identical wells across three independent experiments. Significance denoted by ^∗^ was calculated using Student’s *t*-test with *p* < 0.05. *p*-values were indicated as follows: ^∗^*p* ≤ 0.05; ^∗∗^*p* ≤ 0.01; ^∗∗∗^*p* ≤ 0.001; ^∗∗∗∗^*p* ≤ 0.0001.

We next evaluated whether bacterial clearance was affected by pre-treatment with PGE2 by using a gentamicin protection assay at 2, 24, and 48 hours post infection (hpi). THP-1 cells were pre-treated 1 h before infection with PGE2 (2 μM) or vehicle control (ethanol) and then infected as mentioned previously with either *S.* Typhimurium or *Y. enterocolitica.* PGE2 was maintained in the medium throughout the infection. Although for *S.* Typhimurium infection the effect was not apparent (Figure 4A), in wt *Y. enterocolitica*-infected macrophages, PGE-2 consistently lead to a reduction of the bacterial load at 2, 24, and 48 hpi (Figure 4B), which depended on the dose of PGE2 (Figure 4C). Moreover, the decreased bacterial burden was to some degree also present in HeLa cells (Figure 4D), which also produce inflammasome (Cullen et al., 2015), but are not professional phagocytic cells. At 2 hpi, PGE2 did not have any significant effect on cell cytotoxicity in these infections (Supplementary Figures S2A,B). Finally, PGE2 had no direct effect on bacterial viability (Supplementary Figure S3).

### Regulation of EP2 and EP4 Receptors During Infections With *S.* Typhimurium and *Y. enterocolitica*

**FIGURE 5:**
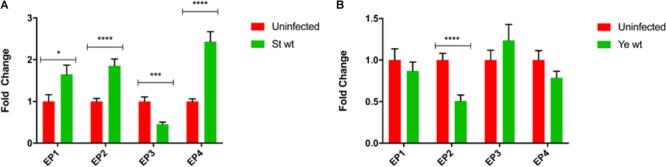
Transcript analysis of PGE2 receptors in THP-1 macrophages during *S.* Typhimurium and *Y. enterocolitica* infection. THP-1 macrophages (2.5 × 10^5^) were infected for 2 h with **(A)** *S.* Typhimurium or **(B)** *Y. enterocolitica* at an MOI of 50:1. Total RNA was extracted using a PureLink RNA mini kit, and cDNA was generated using the iScript system (Bio-Rad). RT-PCR was performed using SYBR green on a CFX96 Real-Time System (Bio-Rad) targeting prostanoid receptors EP1, EP2, EP3, EP4, and GAPDH as a reference gene. Resulting data were then normalized to GAPDH mRNA levels, and uninfected samples served as a baseline for fold change determination using the ΔΔCt method. Resulting data is representative of three biological replicates and three technical replicates and represents the mean fold change ± SD. One-way ANOVA test with Tukey’s multiple testing correction was used to establish statistical significance. *p*-values were indicated as follows: ^∗^*p* ≤ 0.05; ^∗∗^*p* ≤ 0.01; ^∗∗∗^*p* ≤ 0.001; ^∗∗∗∗^*p* ≤ 0.0001.

Prostaglandin E2 exerts its multiple effects by binding to high-affinity G-protein coupled receptors (EP receptors), including EP1, EP2, EP3, and EP4, each with defined downstream targets and effects. Transcripts of all tested PGE2 receptors were present in THP1 macrophages, but upon infection, with *S.* Typhimurium (2 hpi) there was an increase in transcription of EP1, EP2, and EP4 receptors in macrophages, and a decrease in EP3 transcription (Figure 5A). In *Y. enterocolitica* infection, only EP2 receptor was downregulated, and the mRNA levels of remaining PGE2 receptors were not significantly affected (Figure 5B). These data support a possible role for EP2 or EP4 receptors during infections with *S.* Typhimurium and *Y. enterocolitica*.

### EP4 Receptor Signaling Is Responsible for Inflammasome Activation During Infection

**FIGURE 6:**
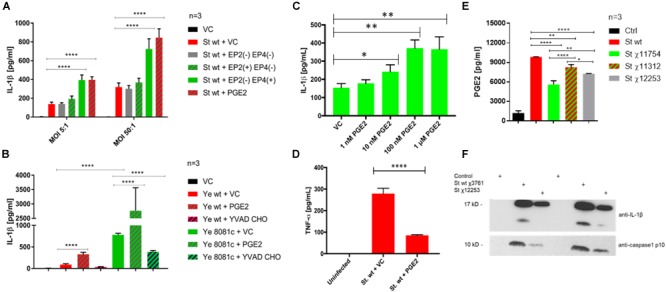
PGE2 signaling and effects on inflammasome activation in *S.* Typhimurium and *Y. enterocolitica* infections. THP-1 macrophages (2.5 × 10^5^) were pre-treated with combinations of PGE2 (2 μM), PF04418948 [EP2(–), EP2 antagonist, 200 nM], L-161,982 [EP4(–), EP4 antagonist, 200 nM], Butaprost [EP2(+), EP2 agonist, 10 μM], L-902,688 [EP4(+), EP4 agonist, 1 μM], or YVAD-CHO (caspase-1 inhibitor, 1 μM) at 2 h prior to infection. The levels of IL-1β **(A–C)** and TNF-α **(D)** in cell culture supernatant from *S. Typhimurium-* **(A,C,D)** or *Y. enterocolitica-* **(B)** infected macrophages were measured *via* ELISA 2 hpi. One-way ANOVA test with Tukey’s multiple testing correction was used to establish statistical significance. *p*-values were indicated as follows: ^∗^*p* ≤ 0.05; ^∗∗^*p* ≤ 0.01; ^∗∗∗^*p* ≤ 0.001; ^∗∗∗∗^*p* ≤ 0.0001. **(E,F)** PGE2 and IL-1β release from THP-1 macrophages infected with *S.* Typhimurium strains. THP-1 cells were infected with indicated strains (Table 1) of wild-type *S.* Typhimurium (MOI 50:1, 2 hpi). PGE2 in cell culture supernatant was measured by Prostaglandin E2 ELISA Kit (Cayman Chemical, United States) and the results were displayed in GraphPad. One-way ANOVA test with Tukey’s multiple testing correction was used to establish statistical significance. *p*-values were indicated as follows: ^∗^*p* ≤ 0.05; ^∗∗^*p* ≤ 0.01; ^∗∗∗^*p* ≤ 0.001; ^∗∗∗∗^*p* ≤ 0.0001. **(E)** Cell culture supernatant was resolved on SDS-PAGE; IL-1β and caspase-1 p10 active form were visualized by western blotting **(F)**.

We hypothesized that there is a positive feedback loop between PGE2 signaling and inflammasome activation, where an increase in inflammasome activity enhances PGE2 release, and inflammasome in turn positively regulates PGE2 production [our study and (Díaz-Muñoz et al., 2012)]. Treatment of cells with PGE2 prior to infection with *S.* Typhimurium or *Y. enterocolitica* did lead to an increase in IL-1β secretion (Figures 6A–C) and a decrease in TNF-α (Figure 6D) secretion. The effect of PGE2 on IL-1β secretion was dose-dependent (Figure 6C) in *S.* Typhimurium infection. To test whether PGE2 signaling *via* EP2 and EP4 receptors results in inflammasome activation during infection, we used a combination of agonists and antagonists selective for EP2 and EP4 receptors. *S.* Typhimurium-infected macrophages treated with EP2 and EP4 antagonists released comparable levels of IL-1β and TNF-α secretion to that of the vehicle control (Figure 6A and Supplementary Figure S4A), whereas treatment with PGE2 (2 μM) resulted in substantially increased levels of IL-1β secretion and decreased levels of TNF-α secretion. The effects on TNF-α secretion can be explained by the fact that an increase in cAMP levels *via* PGE2 or other cAMP agonists leads to TNF-α mRNA destabilization and turnover (Eckmann and Kagnoff, 2001) in a PKA type I-dependent manner (LaRock et al., 2015), and PGE2 therefore also downregulates TNF-α responses. Treatment with the combination of EP2 agonist and EP4 receptor antagonist resulted in a small and not statistically significant increase in IL-1β secretion, but a decreased TNF-α secretion in comparison to the PGE2-treated cells, indicating that EP2-independent pathways activate inflammasome. Use of a combination of EP4 agonist and EP2 antagonist led to altered secretion of IL-1β and TNF-α at comparable levels to the PGE2 treatment. Therefore, signaling *via* the EP4 receptor is most likely responsible for inflammasome activation *via* PGE2 in *S.* Typhimurium infection model. It is unclear whether EP2-/EP4-dependent PKA signaling stimulated inflammasome because two different PKA inhibitors [H-89 and PKI (5-24)] failed to decrease IL-1β release from PGE2-treated and infected macrophages in *S.* Typhimurium or *Y. enterocolitica* infections (data not shown). It is possible that EP4-dependent but PKA-independent pathway is utilized in upregulation of IL-1β release by PKA.

Next, we used a caspase-1 inhibitor to demonstrate that PGE2, which is increased upon inflammasome upregulation (Supplementary Figure S1B), leads to IL-1β release *via* caspase-1 activation, therefore constituting a positive feedback loop. Treatment with the caspase-1 inhibitor YVAD-CHO in combination with PGE2 abolished release of IL-1β upon infection with both *Y. enterocolitica* and *S.* Typhimurium (Figure 6B and Supplementary Figure S4B), supporting the fact that caspase-1 is responsible for the PGE2-triggered stimulation of inflammasome.

*Y. enterocolitica* contains a virulence pYV virulence plasmid (Chung et al., 2016) encoding virulence factors, which lead to the inflammasome activation in infected macrophages. We hypothesized that inflammasome activation during infection with wt *Y. enterocolitica* could be achieved by treating macrophages with PGE2. Indeed, PGE2 triggered a release of IL-1β in THP-1 macrophages infected with wt *Y. enterocolitica* (MOI 50:1, 2 hpi; Figure 6B). Combined treatment with YVAD-CHO caspase-1 inhibitor and PGE2 before infection with *Y. enterocolitica* led to a decreased IL-1β secretion as expected (Figure 6B).

Lipopolysaccharide is known to be a potent co-stimulator of inflammasome activation (Kummari et al., 2015), and it contributes to an increased PGE2 release (Supplementary Figure S1B). To further prove that LPS contributes to PGE2 release as well as the presence of SPI-2, we used *S.* Typhimurium UK-1 mutant strains containing modified LPS structure [χ11312 (Kong et al., 2011) and χ12253 (Liu et al., 2016)], or, as an additional control, we used an attenuated mutant containing a deletion of aroA (χ11754), which is a gene essential to the shikimate pathway (Figures 6E,F). First, we used the anti-PGE2 monoclonal antibody as an alternative way to measure PGE2 levels and confirmed that wt *S.* Typhimurium rapidly increases PGE2 levels at 2 hpi (Figure 6E). *S.* Typhimurium mutants possessing rough LPS (*ΔwaaC41*, χ12253, and *ΔwaaL46*, χ11312; Table 1) or attenuated strain *ΔaroA* (χ11754) lead to a lower PGE2 release in infected macrophages. The *Δ*waaL46 mutant (χ11312) produces complete core-lipid A of LPS with no O-antigen while *ΔwaaC41* (χ12253) has a deficiency in a core lipid A and does not contain O-antigen. The *ΔwaaC41* (χ12253) *S.* Typhimurium used for infection expectedly led to a lower PGE2 release from macrophages in comparison to *ΔwaaL46* mutant (χ11312). However, both, LPS mutants as well as auxotrophic *ΔaroA* strain still led to a production of robust PGE2 release in comparison to uninfected cells. Moreover, in comparison to the infection with wt, χ12253 LPS strain did lead to a lower IL-1β release from cells and decreased the level of caspase-1 cleavage into a mature form (Figure 6F). These pieces of evidence show that intact LPS containing O-antigen and core-lipid A leads to an improved activation of the inflammasome in infected cells and therefore to an increased PGE2 release.

### PGE2 Alters Host Cell Morphology During *S.* Typhimurium Infection

**FIGURE 7:**
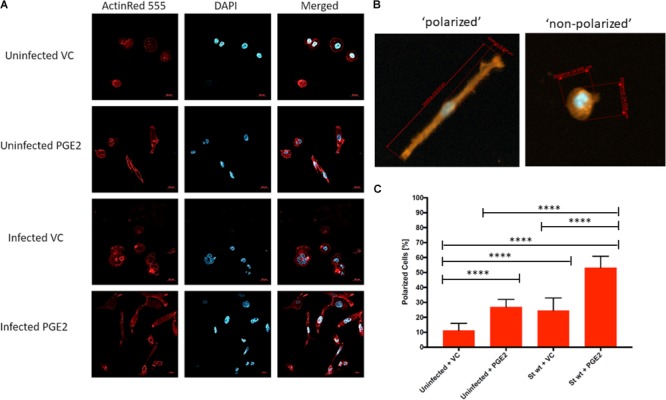
Effect of PGE2 on macrophage morphology in *S.* Typhimurium-infected THP-1 macrophages. THP-1 macrophages (1.5 × 10^6^) were pre-treated with PGE2 (2 μM) or equal (v/v%) concentration of ethanol vehicle control for 2 h before infection with wild-type *S.* Typhimurium (MOI of 50:1, 48 hpi). Cells were then fixed, permeabilized, and stained for the actin cytoskeleton (ActinRed 555, red) and nucleus (DAPI, blue) **(A)**. Polarized cells were determined by having a length to diameter ratio of at least 2:1. Approximately 30 cells were counted from five replicates for a total of 150 cells per treatment (Supplementary Table S1) **(B)**. The number of polarized cells was counted and displayed as a percentage. Approximately 30 cells were counted from five replicates for a total of 150 cells per treatment. ANOVA test with Tukey’s multiple testing correction was used to establish statistical significance **(C)**. *p*-values were indicated as follows: ^∗^*p* ≤ 0.05; ^∗∗^*p* ≤ 0.01; ^∗∗∗^*p* ≤ 0.001; ^∗∗∗∗^*p* ≤ 0.0001.

We next tested the effects of PGE2 on cell morphology, which in macrophages correlates with polarization and activation (McWhorter et al., 2013) that are critical trails of macrophage functionality. We performed light microscopy (24 and 48 hpi) and confocal microscopy studies (48 hpi) of macrophages infected with wt *S.* Typhimurium in presence or absence of exogenous PGE2 (Figure 7 and Supplementary Figure S4C). As compared to the vehicle control, macrophages pre-treated with PGE2 were elongated and formed fiberglass-like appendages (Figure 7A). This altered cell morphology was already present in uninfected cells treated with PGE2, but this effect was further increased upon infection with *S.* Typhimurium, as quantified by cell ratio measurements (Figures 7B,C).

### PGE2 Modulates Macrophage Polarization While Increasing IL-1β and IL-12β p40 Transcription

**FIGURE 8:**
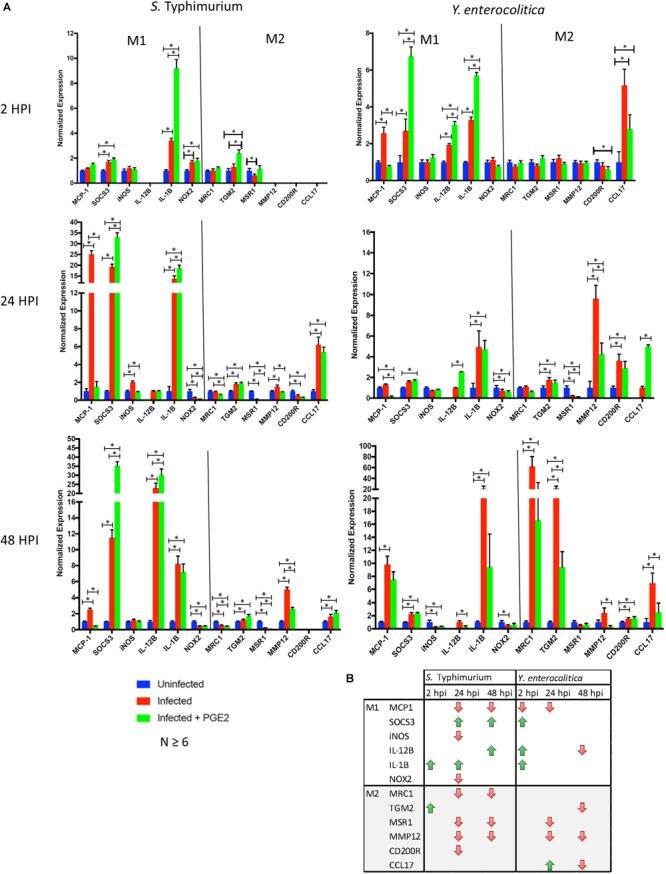
Effects of PGE2 on macrophage polarization. **(A,B)**. THP-1 macrophages were pre-treated with PGE2 (2 μM) or equal (v/v%) concentration of ethanol vehicle control for 2 h before infection with wild-type *S.* Typhimurium or *Y. enterocolitica* (MOI of 50:1, times as displayed in figures). Transcripts analysis of polarization targets in human THP-1 macrophages were measured by RT-(q)PCR (*n* = 6 or higher). Reference genes were determined experimentally for each time point from a set of four potential genes. A single reference gene (RPL37A, GAPDH or TBP) was used for all trials at the indicated time point. **(B)** Down- or upregulation of the transcripts in PGE2-treated infected cells compared to vehicle-treated infected cells is shown. *p*-values were indicated as follows: ^∗^*p* ≤ 0.05; ^∗∗^*p* ≤ 0.01; ^∗∗∗^*p* ≤ 0.001; ^∗∗∗∗^*p* ≤ 0.0001.

We next wanted to confirm whether PGE2-induced changes in macrophage morphology correlate with macrophage polarization, as the association between macrophage morphology and polarization has been previously observed (McWhorter et al., 2013). We analyzed transcripts of genes frequently used as M1 (MCP-1, SOCS3, iNOS, IL-12, IL-1β, IL-12β, and NOX2) or M2 (MRC1, TGM2, MSR1, MMP12, CD200R, and CCL17) macrophage polarization markers in three time points of infection (2, 24, and 48 hpi) and in two different infections (*S*. Typhimurium and *Y. enterocolitica*) of THP-1 macrophages pre-treated with PGE2 or vehicle control (Figure 8).

Prostaglandin E2 contributed to an increased expression level of specific M1 markers in *S.* Typhimurium-infected macrophages (SOCS3 24 and 48 hpi, IL-1β 2 and 24 hpi, and IL-12β 48 hpi; Figure 8, right panel), and downregulation of expression of M2 markers, MRC1, MSR1, and MMP12. Several inconsistencies were noted, including downregulation of MCP-1, iNOS, and NOX2 in PGE2-treated 24 hpi and downregulation of MCP-1 48 hpi. In *Y. enterocolitica*-infected macrophages, PGE2 contributed to an increased expression level of M1 markers (SOCS3 2 hpi, IL-1β 2 hpi, and IL-12β 2 and 24 hpi) and downregulation of M2 markers (CCL17 2 hpi, MMP12, MSR1 24 hpi, and MRC1, TGM2, MMP12, and CCL17 48 hpi). PGE2 also led to a downregulation of M1 markers in *Y. enterocolitica* infection, such as MCP-1 (monocyte chemotactic protein-1; CCL2, 2 hpi, and 24 hpi), and iNOS (at 48 hpi). In summary, the data indicate that PGE2 might shift polarization away from M2 type and that it promotes expression of IL-1β and IL-12β cytokines generally associated with the M1 type. These results are consistent with a decreased bacterial burden in PGE2-treated *Y. enterocolitica*-infected macrophages (Figures 4B,C) as well as, to some degree, HeLa cells (Figure 4D).

## Discussion

Eicosanoids encompass a wide range of bioactive lipids, such as prostaglandins, thromboxanes, leukotrienes, and lipoxins (von Moltke et al., 2012). Biosynthesis of eicosanoids is regulated by PLA-2, which initiates production of AA from phospholipids. Alternatively, the sequential hydrolysis of diacylglycerol and 2-AG endocannabinoid, catalyzed by DAGL and MAGL, respectively, can also yield AA (Nomura et al., 2011; Figure 1). In either case, AA is a substrate for COX-1 and COX-2 enzymes, which catalyze the oxygenation of this polyunsaturated fatty acid into endoperoxide prostaglandin H2 (PGH2). PGH2 is converted to various prostaglandins or thromboxanes by specific prostaglandin or thromboxane synthases (Alhouayek and Muccioli, 2014). While COX-1 enzyme is expressed constitutively in many tissues including monocytes and macrophages, the expression of COX-2 is inducible, and can be triggered by exposure to inflammagens, including interleukin-1 (Mifflin et al., 2002). Since eicosanoids have been shown to regulate inflammation, we measured differential expression of genes responsible for the biosynthesis of these compounds in two different infection models: *S.* Typhimurium and *Y. enterocolitica*. *S.* Typhimurium produces LPS and proteins recognized by PRRs and internal NODD like receptors on macrophages, leading downstream to transcription of pro-inflammatory genes and inflammasome activation, finally followed by secretion of pro-inflammatory cytokines IL-1β and IL-18. Although an increased inflammasome activity is mediated by virulence factors associated with *Salmonella* pathogenicity-1 (SPI-1), such as PrgJ and SipB, or a flagellar protein FliC (LaRock et al., 2015), SPI-2 proteins also lead to inflammasome activation, which is relevant in the clearance of persistent bacteria (Broz and Monack, 2011). On the contrary, specific *Yersinia* effector proteins encoded within its virulence plasmid prevent inflammasome activation, such as effectors YopK (YopQ in *Y. enterocolitica*) (Brodsky et al., 2010) or YopM (LaRock and Cookson, 2012). Therefore, we hypothesized that the presence of the *Y. enterocolitica* virulence plasmid would interfere with the pro-inflammatory eicosanoid production, corresponding to diminished expression of genes regulating this pathway, while the SPI-2 of *S.* Typhimurium would lead to an enhanced eicosanoid production. As expected, COX-2 was significantly upregulated in macrophages infected with both pathogens, although the level of upregulation differed in these infections: *S.* Typhimurium led to four-fold upregulation of COX-2 in comparison to uninfected cells, whereas for *Y. enterocolitica* the upregulation was around twofold relative to uninfected cells.

Interestingly, deletion of the virulence plasmid of *Y. enterocolitica* (8081c) led to a significant upregulation of COX-2 levels, whereas deletion of *ssaV* in *S.* Typhimurium attenuated COX-2 levels by twofold in comparison to wt *S.* Typhimurium (Figure 2). Furthermore, the expression of cPLA-2 phospholipase was induced by wt *S.* Typhimurium infection but not by *Yersinia* infection. Additionally, COX-1 gene expression was downregulated in Δ*ssaV S.* Typhimurium infection and DAGL expression was downregulated by both, wt and Δ*ssaV S.* Typhimurium. Upregulation of COX-2 upon infection was previously shown in the infection model of murine macrophages *S.* Typhimurium (Uchiya and Nikai, 2004). Although COX-2 protein levels were still increased in infected cells, the protein amounts did not completely agree with the transcript data between the wt and mutant strains (Supplementary Figure S1A), and the reason behind this inconsistency could be explained for example by proteolytic events or other post-translational modifications of proteins. However, we also think that these proteins are secreted from *Salmonella* wt-infected cells, similarly as shown for other proteins in prior studies (Anderson and Seilhamer, 1997). The secretion of COX-2 and PLA-2 *via* exosomes was previously demonstrated (Subra et al., 2010), and we have described that exosomes released from *S.* Typhimurium-infected macrophages have an inflammatory effect on naïve cells (Hui et al., 2018). It is likely that the pro-inflammatory effects of exosomes produced by wt *S.* Typhimurium-infected cells could be partially attributed to the presence of COX-2 or PLA-2 in these extracellular vesicles.

The metabolites of COX-2 were also affected by infections (Figure 3). For instance, infection with an SPI-2 mutant of *S.* Typhimurium led to a significant decrease in production of PGF2α, TxB2, and PGE2/PGD2 relative to that seen during wt strain infection. On the other hand, deletion of the virulence plasmid in *Y. enterocolitica* contributed to a significant increase in the levels of AA, TxB2, and PGE2/PGD2 in host cells, relative to those seen during wt strain infection. It was previously demonstrated that the production of PGE2 induced by flagellin injection or *S.* Typhimurium infection was attenuated in cells that do not express NLRC4 (von Moltke et al., 2012), which suggests that the presence of NLRC4 inflammasome is critical for the induction of PGE2 biosynthesis during infection. Although it was previously known that PGE2 is upregulated in cells infected with *S.* Typhimurium (Bowman and Bost, 2004), the upregulation of other eicosanoids in macrophages infected with bacteria is a novel finding, and upregulation of AA is somehow surprising in *Y. enterocolitica* (Figure 3), given that PLA-2 transcript was not significantly upregulated in this infection (Figure 2). We further showed that in addition to SPI-2-based virulence factors, LPS structure and specifically the presence of O-antigen and intact Lipid A are significant in stimulating PGE2 production and inflammasome activation in infected cells (Figures 6E,F).

By using combinations of EP2 and EP4 receptor agonists/antagonists, we showed that while EP2 receptor signaling plays a significant role in regulating TNF-α levels, EP4 receptor stimulation seemed to be the most relevant in the PGE2-mediated secretion of IL-1β upon infection with *S.* Typhimurium (Figure 6). The EP2 and EP4 receptors increase cAMP through their stimulatory G-protein subunit, which consequently activates the enzyme adenylate cyclase converting ATP to cAMP serving as a secondary messenger. For example, classical G-protein coupled receptor stimulation of cAMP conversion from ATP is known to result in binding of cAMP to PKA, dissociation of the regulatory subunits and stimulation of transcription factors such as the phosphorylation of cAMP-responsive binding element (CREB) and alteration of gene transcription (Nomura et al., 2011). It is not unreasonable to suggest that this signaling in the presence of intracellular bacteria could lead to inflammasome activation. However, EP2 and EP4 can stimulate adenylate cyclase differently. The reason we detect EP4 signaling as the PGE2 receptor pathway primarily responsible for inflammasome activation may be due to the higher upregulation of this receptor in macrophages infected with *S.* Typhimurium (Figure 5). We showed that PGE2 effect on IL-1β release in infected cells depends primarily on caspase-1 activity (Figure 6B), but that this PGE2-triggered induction of IL-1β release is not associated with higher cytotoxicity at 2 hpi (Supplementary Figure S2), which could suggest alternative inflammasome activation by PGE2 (Gaidt et al., 2016).

Prostaglandin E2-treated macrophages had a reduced bacterial load in *Y. enterocolitica* infection, but not *S.* Typhimurium (Figure 4). Our earlier results showed that PGE2 increases phagocytosis of zymosan particles in HD11 macrophages (Lee et al., 2017); therefore, we suspect that increased phagocytosis could be responsible for this reduced bacterial load. Other explanations of this phenomenon include differential activation of macrophages exposed to a high PGE2 dose. PGE2-treated and *S.* Typhimurium-infected cells underwent significant changes in morphology (Figure 7) and altered expression of genes regulating M1/M2 polarization (Figure 8). Infection with *S.* Typhimurium generally stimulates LPS-dependent M1 macrophage profile in murine macrophages (Mifflin et al., 2002) while PGE2 is associated with M2 polarization profiles, for example, *via* cyclic-AMP responsive element binding (CREB) induction of Krupple-like factor 4 (KLF4) (Broz and Monack, 2011). However, in our study, an exogenous addition of PGE2 appeared to modulate shift in M1/M2 phenotype away from M2 phenotype in response to *S.* Typhimurium infection (Figure 8), at least based on the stimulation of select M1 genes/proteins by PGE2, for instance, IL-1β and IL-23/17.

Further investigation is required to establish how PGE2 shapes the macrophage polarization upon *S.* Typhimurium and *Y. enterocolitica* infections. We suggest that PGE2 might stimulate the killing of bacteria by macrophages in certain infections (*Y. enterocolitica*) but not in others (*S.* Typhimurium). Other possible roles of PGE2 in these infection models are the attraction of neutrophils to the site of infection. It has recently been reported that eicosanoid biosynthesis plays a role in the efferocytosis of neighboring neutrophils in mice (Jorgensen et al., 2016), where PGE2 could contribute toward the mechanism of neutrophil migration. Regarding other limitations of our work, we have not identified specific effector proteins encoded in SPI-2 of *S.* Typhimurium, or the virulence plasmid of *Y. enterocolitica*, which might regulate the release of particular eicosanoids from infected cells or affect the enzymes involved in the eicosanoid biosynthesis. However, in *Yersinia*, the suspected effectors are the ones which regulate inflammasome, including YopM and YopQ/K (Brodsky et al., 2010; LaRock and Cookson, 2012; Chung et al., 2016), while in *S.* Typhimurium, one of the suspected effectors is SpiC (Uchiya and Nikai, 2004).

**FIGURE 9:**
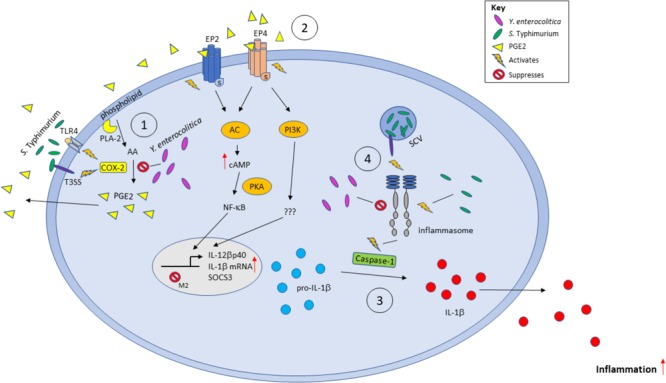
Model of the effects of PGE2 secretion during infection with Gram-negative bacteria *S.* Typhimurium and *Y. enterocolitica*. Bacterial pathogens can stimulate the production of PGE2 from AA by activating COX-2 *via* TLR-LPS signaling or through other virulence factors such as *Salmonella* SpiC or *Yersinia* YopM. PGE2 is secreted and acts locally on EP2/EP4 receptors to repress M2 macrophage gene transcription by SOCS3. PGE2 increases IL-1β and IL-12βp40 transcription. The immature IL-1β is converted to mature IL-1β by activated caspase-1 during inflammasome activation and secreted into the environment. Inflammasomes can be stimulated or inhibited by specific bacterial components and virulence factors such as bacterial flagellin, or by YopM/SopB in *Y. enterocolitica* and *S.* Typhimurium, respectively.

In summary (Figure 9), our current work provides several lines of evidence that PGE2 eicosanoid enhances inflammasome activation upon bacterial infection, leading to changes in macrophage polarization and function. However, implications of these PGE2-mediated changes of macrophage function and their role in the clearance of bacteria should be further addressed by appropriate *in vivo* studies.

## Author Contributions

ME, MR, AS, and AW contributed in conceptualization. ME, MR, AS, LM, and EK contributed in writing. ME, EK, AS, AR, LM, WH, JL, and AB contributed in investigation. ME contributed in funding acquisition. AS and JL contributed in formal analysis. MR, ME, AR, AS, EK, and AW contributed in methodology. MR and ME contributed in supervision. MR and ME contributed in resources. AS and AW contributed in validation. AS, ME, and AW contributed in visualization.

## Conflict of Interest Statement

The authors declare that the research was conducted in the absence of any commercial or financial relationships that could be construed as a potential conflict of interest.

## References

[B1] Alhouayek M. Muccioli G. G. (2014). COX-2-derived endocannabinoid metabolites as novel inflammatory mediators. *Trends Pharmacol. Sci.* 35 284–292. 10.1016/j.tips.2014.03.001 24684963

[B2] Alugubelly N. Hercik K. Kibler P. Nanduri B. Edelmann M. J. (2016). Analysis of differentially expressed proteins in Yersinia enterocolitica-infected HeLa cells. *Biochim. Biophys. Acta* 1864 562–569. 10.1016/j.bbapap.2016.02.004 26854600 PMC5505629

[B3] Anderson L. Seilhamer J. (1997). A comparison of selected mRNA and protein abundances in human liver. *Electrophoresis* 18 533–537. 10.1002/elps.1150180333 9150937

[B4] Antunes L. C. Arena E. T. Menendez A. Han J. Ferreira R. B. Buckner M. M. (2011). Impact of *Salmonella* infection on host hormone metabolism revealed by metabolomics. *Infect Immun.* 79 1759–1769. 10.1128/IAI.01373-10 21321075 PMC3067560

[B5] Beuzón C. R. Méresse S. Unsworth K. E. Ruíz-Albert J. Garvis S. Waterman S. R. (2000). *Salmonella* maintains the integrity of its intracellular vacuole through the action of SifA. *EMBO J.* 19 3235–3249. 10.1093/emboj/19.13.3235 10880437 PMC313946

[B6] Bowman C. C. Bost K. L. (2004). Cyclooxygenase-2-mediated prostaglandin E2 production in mesenteric lymph nodes and in cultured macrophages and dendritic cells after infection with *Salmonella*. *J. Immunol.* 172 2469–2475. 10.4049/jimmunol.172.4.2469 14764719

[B7] Brodsky I. E. Palm N. W. Sadanand S. Ryndak M. B. Sutterwala F. S. Flavell R. A. (2010). A Yersinia effector protein promotes virulence by preventing inflammasome recognition of the type III secretion system. *Cell Host Microbe* 7 376–387. 10.1016/j.chom.2010.04.009 20478539 PMC2883865

[B8] Broz P. Monack D. M. (2011). Molecular mechanisms of inflammasome activation during microbial infections. *Immunol. Rev.* 243 174–190. 10.1111/j.1600-065X.2011.01041.x 21884176 PMC3170129

[B9] Buckner M. M. Antunes L. C. Gill N. Russell S. L. Shames S. R. Finlay B. B. (2013). 15-Deoxy-Δ12,14-prostaglandin J2 inhibits macrophage colonization by *Salmonella enterica* serovar Typhimurium. *PLoS One* 8:e69759. 10.1371/journal.pone.0069759 23922794 PMC3724865

[B10] Chung L. K. Park Y. H. Zheng Y. Brodsky I. E. Hearing P. Kastner D. L. (2016). The yersinia virulence factor yopm hijacks host kinases to inhibit type III effector-triggered activation of the pyrin inflammasome. *Cell Host Microbe* 20 296–306. 10.1016/j.chom.2016.07.018 27569559 PMC5025386

[B11] Crump J. A. Sjölund-Karlsson M. Gordon M. A. Parry C. M. (2015). Epidemiology, clinical presentation, laboratory diagnosis, antimicrobial resistance, and antimicrobial management of invasive *Salmonella* infections. *Clin. Microbiol. Rev.* 28 901–937. 10.1128/CMR.00002-15 26180063 PMC4503790

[B12] Cullen S. P. Kearney C. J. Clancy D. M. Martin S. J. (2015). Diverse activators of the NLRP3 inflammasome promote IL-1β secretion by triggering necrosis. *Cell Rep.* 11 1535–1548. 10.1016/j.celrep.2015.05.003 26027935

[B13] Deatherage Kaiser B. L. Li J. Sanford J. A. Kim Y. M. Kronewitter S. R. Jones M. B. (2013). A multi-omic view of host-pathogen-commensal interplay in *Salmonella*-mediated intestinal infection. *PLoS One* 8:e67155. 10.1371/journal.pone.0067155 23840608 PMC3694140

[B14] Dennis E. A. Norris P. C. (2015). Eicosanoid storm in infection and inflammation. *Nat. Rev. Immunol.* 15 511–523. 10.1038/nri3859 26139350 PMC4606863

[B15] Díaz-Muñoz M. D. Osma-García I. C. Fresno M. Iñiguez M. A. (2012). Involvement of PGE2 and the cAMP signalling pathway in the up-regulation of COX-2 and mPGES-1 expression in LPS-activated macrophages. *Biochem. J.* 443 451–461. 10.1042/BJ20111052 22268508

[B16] Eckmann L. Kagnoff M. F. (2001). Cytokines in host defense against *Salmonella*. *Microbes Infect.* 3 1191–1200. 10.1016/S1286-4579(01)01479-4 11755407

[B17] Edelmann M. J. Kramer H. B. Altun M. Kessler B. M. (2010). Post-translational modification of the deubiquitinating enzyme otubain 1 modulates active RhoA levels and susceptibility to Yersinia invasion. *FEBS J.* 277 2515–2530. 10.1111/j.1742-4658.2010.07665.x 20553488

[B18] Funk C. D. (2001). Prostaglandins and leukotrienes: advances in eicosanoid biology. *Science* 294 1871–1875. 10.1126/science.294.5548.1871 11729303

[B19] Gaidt M. M. Ebert T. S. Chauhan D. Schmidt T. Schmid-Burgk J. L. Rapino F. (2016). Human monocytes engage an alternative inflammasome pathway. *Immunity* 44 833–846. 10.1016/j.immuni.2016.01.012 27037191

[B20] Hassan J. O. Curtiss R. (1990). Control of colonization by virulent *Salmonella* typhimurium by oral immunization of chickens with avirulent delta cya delta crp S. typhimurium. *Res. Microbiol.* 141 839–850. 10.1016/0923-2508(90)90119-B 2101473

[B21] Hoffmann S. Anekwe T. D. (2013). *United States. Department of Agriculture. Economic Research Service. Making sense of recent cost-of-foodborne-illness estimates.* Available at: https://www.ers.usda.gov/

[B22] Hui W. W. Hercik K. Belsare S. Alugubelly N. Clapp B. Rinaldi C. (2018). *Salmonella enterica* serovar Typhimurium alters the extracellular proteome of macrophages and leads to the production of proinflammatory exosomes. *Infect. Immun.* 86:e00386-17. 10.1128/IAI.00386-17 29158431 PMC5778363

[B23] Jackstadt R. Sansom O. J. (2017). The Wae to repair: prostaglandin E2 (PGE2) triggers intestinal wound repair. *EMBO J.* 36 3–4. 10.15252/embj.201695973 27932445 PMC5210127

[B24] Jorgensen I. Lopez J. P. Laufer S. A. Miao E. A. (2016). IL-1β, IL-18, and eicosanoids promote neutrophil recruitment to pore-induced intracellular traps following pyroptosis. *Eur. J. Immunol.* 46 2761–2766. 10.1002/eji.201646647 27682622 PMC5138142

[B25] Kong Q. Yang J. Liu Q. Alamuri P. Roland K. L. Curtiss R. (2011). Effect of deletion of genes involved in lipopolysaccharide core and O-antigen synthesis on virulence and immunogenicity of *Salmonella enterica* serovar typhimurium. *Infect. Immun.* 79 4227–4239. 10.1128/IAI.05398-11 21768282 PMC3187260

[B26] Kummari E. Alugubelly N. Hsu C. Y. Dong B. Nanduri B. Edelmann M. J. (2015). Activity-based proteomic profiling of deubiquitinating enzymes in *Salmonella*-infected macrophages leads to identification of putative function of UCH-L5 in inflammasome regulation. *PLoS One* 10:e0135531. 10.1371/journal.pone.0135531 26267804 PMC4534353

[B27] Lämmermann T. Afonso P. V. Angermann B. R. Wang J. M. Kastenmüller W. Parent C. A. (2013). Neutrophil swarms require LTB4 and integrins at sites of cell death in vivo. *Nature* 498 371–375. 10.1038/nature12175 23708969 PMC3879961

[B28] LaRock C. N. Cookson B. T. (2012). The Yersinia virulence effector YopM binds caspase-1 to arrest inflammasome assembly and processing. *Cell Host Microbe* 12 799–805. 10.1016/j.chom.2012.10.020 23245324 PMC3703949

[B29] LaRock D. L. Chaudhary A. Miller S. I. (2015). *Salmonellae* interactions with host processes. *Nat. Rev. Microbiol.* 13 191–205. 10.1038/nrmicro3420 25749450 PMC5074537

[B30] Lee J. H. Hou X. Kummari E. Borazjani A. Edelmann M. J. Ross M. K. (2017). Endocannabinoid hydrolases in avian HD11 macrophages identified by chemoproteomics: inactivation by small-molecule inhibitors and pathogen-induced downregulation of their activity. *Mol. Cell Biochem.* 444 125–141. 10.1007/s11010-017-3237-0 29196970

[B31] Liu Q. Yi J. Liang K. Liu T. Roland K. L. Jiang Y. (2016). Outer membrane vesicles derived from *Salmonella* Typhimurium mutants with truncated LPS induce cross-protective immune responses against infection of *Salmonella enterica* serovars in the mouse model. *Int. J. Med. Microbiol.* 306 697–706. 10.1016/j.ijmm.2016.08.004 27578609 PMC5206754

[B32] McWhorter F. Y. Wang T. Nguyen P. Chung T. Liu W. F. (2013). Modulation of macrophage phenotype by cell shape. *Proc. Natl. Acad. Sci. U.S.A.* 110 17253–17258. 10.1073/pnas.1308887110 24101477 PMC3808615

[B33] Mifflin R. C. Saada J. I. Di Mari J. F. Adegboyega P. A. Valentich J. D. Powell D. W. (2002). Regulation of COX-2 expression in human intestinal myofibroblasts: mechanisms of IL-1-mediated induction. *Am. J. Physiol. Cell Physiol.* 282 C824–C834. 10.1152/ajpcell.00388.2001 11880271

[B34] Nomura D. K. Morrison B. E. Blankman J. L. Long J. Z. Kinsey S. G. Marcondes M. C. (2011). Endocannabinoid hydrolysis generates brain prostaglandins that promote neuroinflammation. *Science* 334 809–813. 10.1126/science.1209200 22021672 PMC3249428

[B35] Portnoy D. A. Moseley S. L. Falkow S. (1981). Characterization of plasmids and plasmid-associated determinants of Yersinia enterocolitica pathogenesis. *Infect. Immun.* 31 775–782. 7216474 10.1128/iai.31.2.775-782.1981 PMC351377

[B36] Rauch I. Deets K. A. Ji D. X. von Moltke J. Tenthorey J. L. Lee A. Y. (2017). NAIP-NLRC4 inflammasomes coordinate intestinal epithelial cell expulsion with eicosanoid and IL-18 release via activation of caspase-1 and -8. *Immunity* 46 649–659. 10.1016/j.immuni.2017.03.016 28410991 PMC5476318

[B37] Scharff R. L. (2012). Economic burden from health losses due to foodborne illness in the United States. *J. Food Prot.* 75 123–131. 10.4315/0362-028X.JFP-11-058 22221364

[B38] Schmittgen T. D. Livak K. J. (2008). Analyzing real-time PCR data by the comparative C(T) method. *Nat. Protoc.* 3 1101–1108. 10.1038/nprot.2008.73 18546601

[B39] Subra C. Grand D. Laulagnier K. Stella A. Lambeau G. Paillasse M. (2010). Exosomes account for vesicle-mediated transcellular transport of activatable phospholipases and prostaglandins. *J. Lipid Res.* 51 2105–2120. 10.1194/jlr.M003657 20424270 PMC2903822

[B40] Tanioka T. Nakatani Y. Semmyo N. Murakami M. Kudo I. (2000). Molecular identification of cytosolic prostaglandin E2 synthase that is functionally coupled with cyclooxygenase-1 in immediate prostaglandin E2 biosynthesis. *J. Biol. Chem.* 275 32775–32782. 10.1074/jbc.M003504200 10922363

[B41] Tyrkalska S. D. Candel S. Angosto D. Gómez-Abellán V. Martín-Sánchez F. García-Moreno D. (2016). Neutrophils mediate *Salmonella* Typhimurium clearance through the GBP4 inflammasome-dependent production of prostaglandins. *Nat. Commun.* 7:12077. 10.1038/ncomms12077 27363812 PMC4932187

[B42] Uchiya K. Nikai T. (2004). *Salmonella enterica* serovar Typhimurium infection induces cyclooxygenase 2 expression in macrophages: involvement of *Salmonella* pathogenicity island 2. *Infect. Immun.* 72 6860–6869. 10.1128/IAI.72.12.6860-6869 15557607 PMC529151

[B43] von Moltke J. Trinidad N. J. Moayeri M. Kintzer A. F. Wang S. B. van Rooijen N. (2012). Rapid induction of inflammatory lipid mediators by the inflammasome in vivo. *Nature* 490 107–111. 10.1038/nature11351 22902502 PMC3465483

[B44] Winter S. E. Winter M. G. Atluri V. Poon V. Romão E. L. Tsolis R. M. (2015). The flagellar regulator TviA reduces pyroptosis by *Salmonella enterica* serovar Typhi. *Infect. Immun.* 83 1546–1555. 10.1128/IAI.02803-14 25644011 PMC4363433

[B45] Zajac E. Schweighofer B. Kupriyanova T. A. Juncker-Jensen A. Minder P. Quigley J. P. (2013). Angiogenic capacity of M1- and M2-polarized macrophages is determined by the levels of TIMP-1 complexed with their secreted proMMP-9. *Blood* 122 4054–4067. 10.1182/blood-2013-05-501494 24174628 PMC3862278

[B46] Zhuang Y. Zhao F. Liang J. Deng X. Zhang Y. Ding G. (2017). Activation of COX-2/mPGES-1/PGE2 Cascade via NLRP3 inflammasome contributes to albumin-induced proximal tubule cell injury. *Cell. Physiol. Biochem.* 42 797–807. 10.1159/000478070 28628921

