# Supplementary material for: PGE2 Augments Inflammasome Activation and M1 Polarization in Macrophages Infected With Salmonella Typhimurium and Yersinia enterocolitica
Source: Front Microbiol. 2018 Oct 31;9:2447. doi: 10.3389/fmicb.2018.02447 (PMC6220063; doi:10.3389/fmicb.2018.02447)
Supplement: Supplementary file 1 [file Data_Sheet_1.docx]

**Supplementary figure legends**

**Supplementary Figure 1. Levels of eicosanoids secreted into culture medium upon infection with *S.* Typhimurium** **or inflammasome activation and effect of b-AP15 on eicosanoid production. (A)** THP-1 cells were infected with S. Typhimurium for 2 hours at an MOI 50:1. The cells were lysed, and protein samples were separated by SDS-PAGE, followed by anti-COX2, anti-PLA-2, and anti-beta actin western blotting (loading control).  **(B).** THP-1 cells were pre-treated with 1 μM b-AP15 (or vehicle control, DMSO) for 15 minutes (b-AP15 is a UCHL5/UCH14 inhibitor found to inhibit inflammasome [1]), and infected with wild-type *S.* Typhimurium for 2 hrs (MOI 50:1). Media were collected, and prostaglandins were obtained by using a solid-phase extraction method. The media were analyzed for AA, TxB2, and PGE2/PGD_2_ by using Triple Quadrupole mass spectrometry. Relative abundance was calculated using internal standards. One-way ANOVA test with Tukey’s multiple testing correction was used to establish statistical significance. P-values were indicated as follows: * p≤ 0.05; ** p ≤ 0.01; *** p≤ 0.001; **** p ≤ 0.0001. **(C).** THP-1 cells were treated with *S.* Typhimurium LPS (1 μg/ml; Sigma-Aldrich) for 4 hours, followed by treatment with 10 μM nigericin for 1 hour to activate inflammasome. TxB2 and PGE2/PGD_2_ in cell culture media were analyzed by using Triple Quadrupole mass spectrometry as described in **(A)**.

**Supplementary Figure 2. PGE2 effects on cytotoxicity upon infection with *S.* Typhimurium or *Y. enterocolitica* 2 hpi.** THP-1 macrophages were pre-treated with 2 (mM) PGE2 or equal (v/v%) concentration of ethanol vehicle control and infected with *S.*Typhimurium (A) or *Y. enterocolitica* (B) for 2 hours and then cytotoxicity was assessed using a Pierce LDH Cytotoxicity assay kit. Percent release of LDH is compared to that of the max release control and indicates no significant differences between infected vehicle-treated and infected PGE2-treated cells.

**Supplementary Figure 3.** **PGE2 effect on bacterial growth.** 20 mL of LB culture was inoculated from an ON culture to an initial OD of 0.05 in the presence of PGE2 (2mM) or an equal concentration (v/v%) of ethanol vehicle control. OD600 was measured every hour for 8 hours by a spectrophotometer and plotted over time. A final stationary phase time point was taken after 24 hours of growth. N = 3.

**Supplementary Figure 4.** **(A).** THP-1 macrophages (2.5 x 10^5^) were pre-treated with combinations of PGE2 (2 µM), PF04418948 [EP2(-), EP2 antagonist, 200 nM], L-161,982 [EP4(-), EP4 antagonist, 200 nM], Butaprost [EP2(+), EP2 agonist, 10 µM], L-902,688 [EP4(+), EP4 agonist, 1 µM], at 2 hours prior to infection. The levels of TNF-α in cell culture supernatant from *S.* Typhimurium-infected macrophages were measured via ELISA 2 hpi. One-way ANOVA test with Tukey’s multiple testing correction was used to establish statistical significance. P-values were indicated as follows: * p≤ 0.05; ** p ≤ 0.01; *** p≤ 0.001; **** p ≤ 0.0001. **(B).** **Effect of the caspase-1 inhibitor on PGE2-induced effect on IL-1 β release.** THP-1 macrophages (2.5 x 10^5^) were pre-treated with combinations of PGE2 (2 µM) and YVAD-CHO (caspase-1 inhibitor, 1 µM) for 2 hours before a 2-hour infection. The levels of IL-1β cell culture supernatant from *S.* Typhimurium or *Y. enterocolitica*-infected macrophages were measured by using ELISA. One-way ANOVA test with Tukey’s multiple testing correction was used to establish statistical significance. P-values were indicated as follows: * p≤ 0.05; ** p ≤ 0.01; *** p≤ 0.001; **** p ≤ 0.0001. **(C).** THP-1 macrophages were grown on coverslips and pre-treated with PGE2 (2 µM) or vehicle for 2 hours before a 24-hour and 48-hour infection with *S.* Typhimurium (MOI 50:1). Such treated cells were visualized by light microscopy.

**Supplementary tables**

**Supplementary Table 1.** **PGE2 induces macrophage polarization in *S.* Typhimurium-infected THP-1 macrophages.** THP-1 macrophages (1.5 x 10^6^) were pre-treated with PGE2 (2 µM) or equal (v/v %) concentration of ethanol vehicle control for two hours before infection with wild-type *S.* Typhimurium (MOI of 50:1, 48 hpi). Cells were then fixed, permeabilized, and stained for the actin cytoskeleton (red) and nucleus (blue). The polarized cells were counted and the percentage calculated. Polarized cells were determined by having a length to diameter ratio of at least 2:1. Approximately 30 cells were counted from 5 replicates for a total of 150 cells per treatment.

|  |  |  |  |  |  |  |  |
| --- | --- | --- | --- | --- | --- | --- | --- |
| **uninfected - control** | **Un VC1** | **Un VC2** | **Un VC3** | **Un VC4** | **Un VC5** | **Total** | **Error** |
| Polarized | 4.00 | 4.00 | 2.00 | 4.00 | 3.00 | 17.00 |  |
| Unpolarized | 16.00 | 25.00 | 31.00 | 38.00 | 24.00 | 134.00 |  |
| Percent Polarized | 20.00 | 13.79 | 6.06 | 9.52 | 11.11 | **11.26** | 4.69 |
| Total Cells Counted |  |  |  |  |  | 151.00 |  |
|  |  |  |  |  |  |  |  |
| **uninfected - PGE2** | **Un PG1** | **Un PG2** | **Un PG3** | **Un PG4** | **Un PG5** | **Total** | **Error** |
| Polarized | 7.00 | 9.00 | 7.00 | 6.00 | 11.00 | 40.00 |  |
| Unpolarized | 22.00 | 25.00 | 23.00 | 18.00 | 20.00 | 108.00 |  |
| Percent Polarized | 24.14 | 26.47 | 23.33 | 25.00 | 35.48 | **27.03** | 4.95 |
| Total Cells Counted |  |  |  |  |  | 148.00 |  |
|  |  |  |  |  |  |  |  |
| **infected - control** | **Inf VC1** | **Inf VC2** | **Inf VC3** | **Inf VC4** | **Inf VC5** | **Total** | **Error** |
| Polarized | 8.00 | 10.00 | 3.00 | 11.00 | 8.00 | 40.00 |  |
| Unpolarized | 15.00 | 32.00 | 22.00 | 27.00 | 27.00 | 123.00 |  |
| Percent Polarized | 34.78 | 23.81 | 12.00 | 28.95 | 22.86 | **24.54** | 8.44 |
| Total Cells Counted |  |  |  |  |  | 163.00 |  |
|  |  |  |  |  |  |  |  |
| **infected- PGE2** | **Inf PG1** | **Inf PG2** | **Inf PG3** | **Inf PG4** | **Inf PG5** | **Total** | **Error** |
| Polarized | 20.00 | 21.00 | 21.00 | 9.00 | 11.00 | 82.00 |  |
| Unpolarized | 14.00 | 16.00 | 16.00 | 11.00 | 15.00 | 72.00 |  |
| Percent Polarized | 58.82 | 56.76 | 56.76 | 45.00 | 42.31 | **53.25** | 7.66 |
| Total Cells Counted |  |  |  |  |  | 154.00 |  |

**Supplementary References:**

1. Kummari E, Alugubelly N, Hsu CY, Dong B, Nanduri B, Edelmann MJ. Activity-Based Proteomic Profiling of Deubiquitinating Enzymes in Salmonella-Infected Macrophages Leads to Identification of Putative Function of UCH-L5 in Inflammasome Regulation. PLoS One. 2015;10(8):e0135531. doi: 10.1371/journal.pone.0135531. PubMed PMID: 26267804; PubMed Central PMCID: PMCPMC4534353.
