# Supplementary figures and images for: PGE2 Augments Inflammasome Activation and M1 Polarization in Macrophages Infected With Salmonella Typhimurium and Yersinia enterocolitica
Source: Front Microbiol. 2018 Oct 31;9:2447. doi: 10.3389/fmicb.2018.02447 (PMC6220063; doi:10.3389/fmicb.2018.02447)

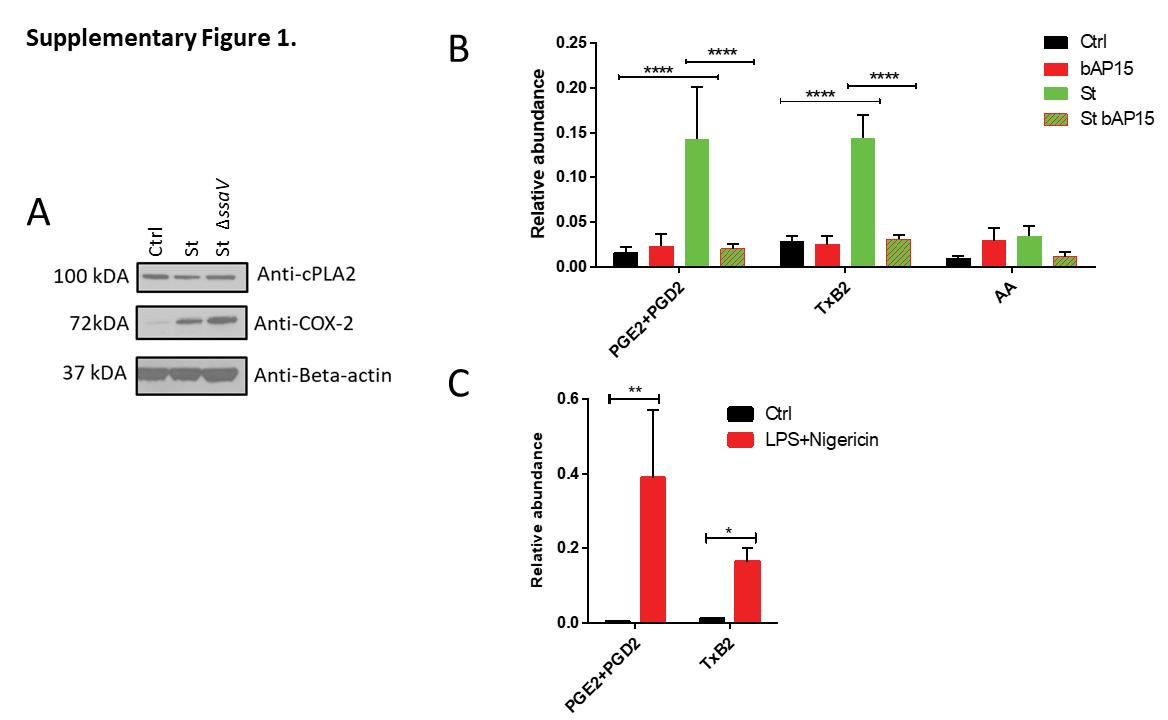

Supplement: Supplementary file 2 [file Image_1.tif]

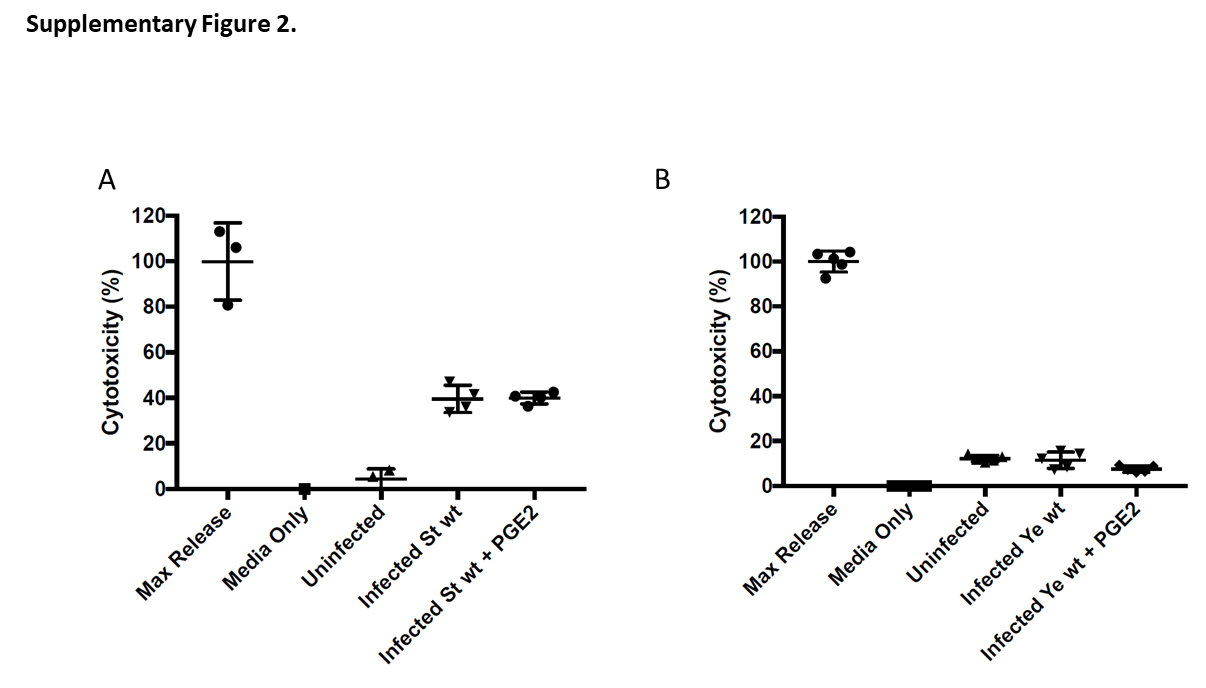

Supplement: Supplementary file 3 [file Image_2.tif]

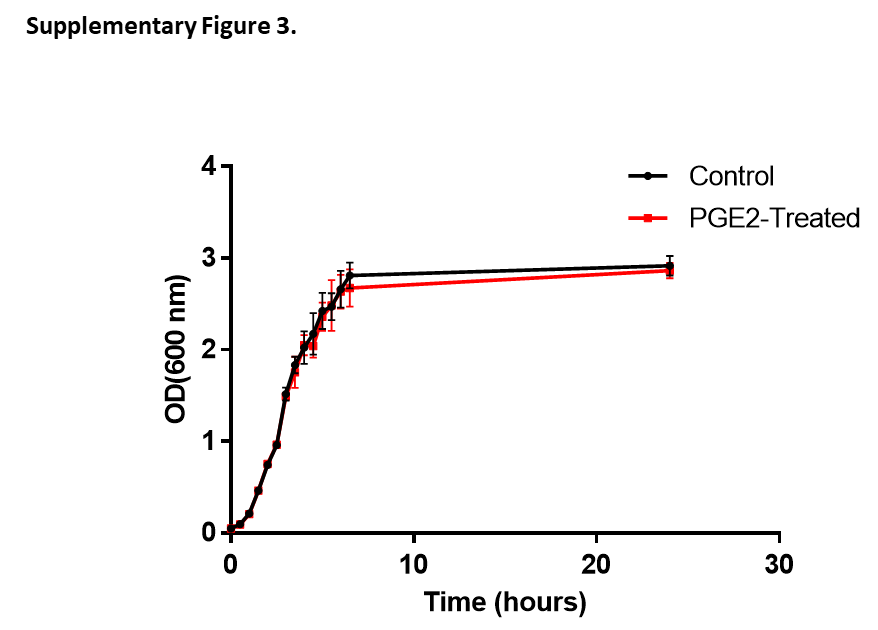

Supplement: Supplementary file 4 [file Image_3.tif]

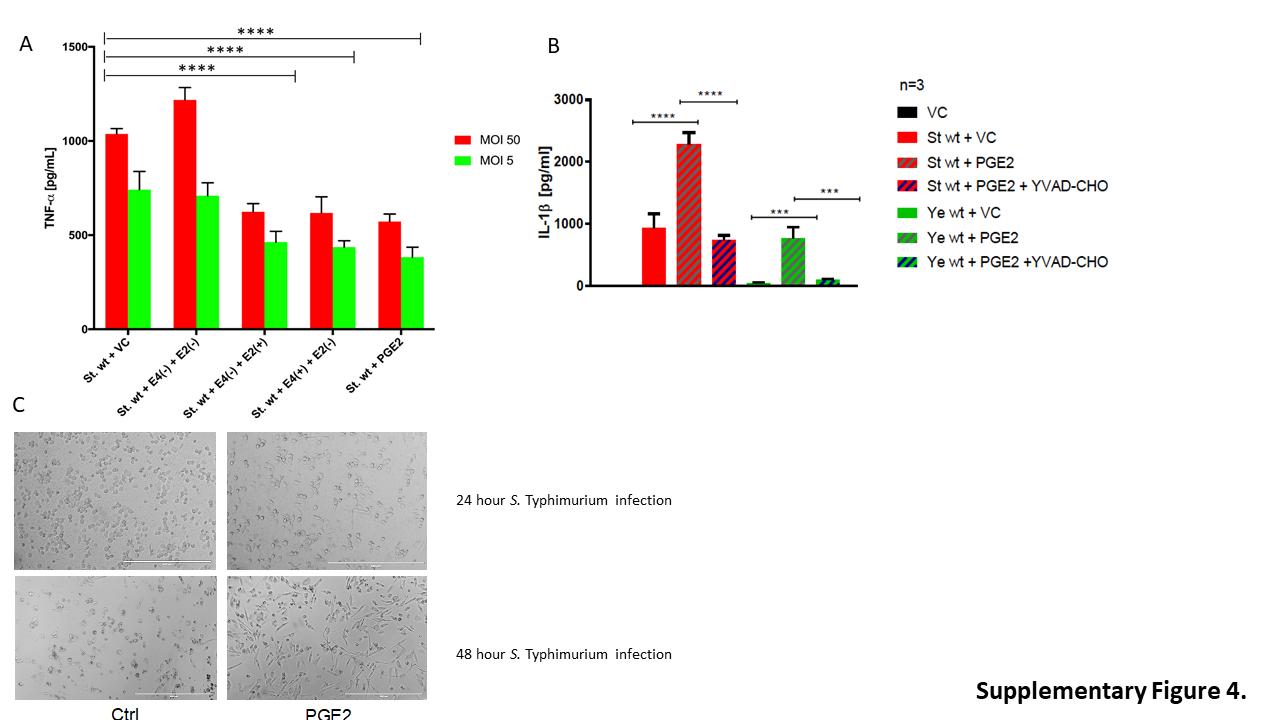

Supplement: Supplementary file 5 [file Image_4.tif]
